# Facilitating health promoting ideas and actions: participatory research in an underserved Swedish residential area

**DOI:** 10.1186/s12889-021-12431-y

**Published:** 2022-01-24

**Authors:** Maria Magnusson, Lisa M. Vaughn, Katharina Wretlind, Heléne Bertéus Forslund, Christina Berg

**Affiliations:** 1 Public Health Unit, Angered Hospital/SV Hospital Group, Region Västra Götaland, Box 63, 424 22 Angered, Sweden; 2 Department of Pediatrics, University of Cincinnati College of Medicine/Cincinnati Children’s Hospital Medical Center, Cincinnati, OH 3333 Burnet Ave, 45220 USA; 3 Public Dental Service Västra Götaland, Region Västra Götaland, Regionens hus, 405 44 Gothenburg, Sweden; 4 Department of Internal Medicine and Clinical Nutrition, Sahlgrenska Academy, University of Gothenburg, Box 459, 413 45 Gothenburg, Sweden; 5 Department of Food and Nutrition, and Sport Science, University of Gothenburg, Box 100, 405 30 Gothenburg, Sweden

**Keywords:** Community-based participatory research, Community-academic partnership, Empowerment, Communication, Health literacy

## Abstract

**Background:**

For an intervention to contribute to decreased health gaps, people living in underserved areas must participate in the research-to-action process during the development of the intervention. The purpose of this study was to collaborate with residents living in a Swedish underserved area to generate health and wellness priorities and actions.

**Methods:**

We applied Group Level Assessment (GLA) together with people living in a Swedish neighborhood where obesity, dental caries and other illnesses are prevalent. GLA is a qualitative, participatory methodology that is designed for a large group to generate and evaluate relevant needs and priorities within a lens of action for positive social change. Residents were recruited by posters, postcards and snowball sampling. In total, 47 residents participated. Eight GLA sessions were held over a five-month time period.

**Results:**

The GLA sessions resulted in reflections, proposals and actions for change by the residents. Adolescent and parent need for support, improved communication and more meeting places were highlighted as priorities for promoting health and well-being. The results were presented for stakeholders in a report and an exhibition and some of the participants started a language café.

**Conclusions:**

GLA emphasised the participants’ perspective. The participatory process helped them identify what they thought valuable and relevant concerning health issues and supported them in taking actions to achieve change.

## Introduction

Swedish public health, measured by means such as obesity or premature death, is generally good [1], but health inequalities remain. People in groups with short education have lower life expectancy and higher mortality compared with those with post-secondary education. The same imbalance shows for violence-related injuries, obesity and self-rated health [2–4].

Worse health is more common in underserved areas characterized by marginalization and discrimination. Such neighbourhoods are, with the words of Loic Wacquant, being territorially stigmatized: “A *blemish of place* is thus super-imposed on the already existing stigma traditionally associated with poverty and ethnic origin or postcolonial immigrant status” (p 67) [5]. The concept has been applied also in Swedish studies [6]. Analysis by Scarpa indicate that the residential segregation and the general widening of the gap in living conditions in Sweden to a large extent is driven by increase in income inequality [7].

We need more knowledge on how to reduce health inequalities [8] and contrary to what is desired, public health interventions may unfortunately widen the gap [9]. One reason for this is that staff who plan and conduct such interventions commonly differ from people who are the target of the interventions most notably by social position and economic circumstances, thus being more distant from or unaware of perspectives and needs in these groups. Another reason is that health interventions often draw exclusively on academically produced knowledge and do not reflect the lived expertise and collective wisdom of insiders [10, 11].

To overcome this problem, different approaches that attempt to enable control, influence and participation from people in groups with less favorable social and economic positions and in different cultural contexts are necessary. Therefore, community-level efforts aiming at increase peoples’ capability and confidence to engage in collective actions for their well-being and health are important [12]. Participatory research methods that amplify and garner community participation have been developed within the action-oriented and community-partnered alternative research paradigm community-based participatory research (CBPR). In CBPR, community members are involved in the development of research questions, collection and interpretation of data, dissemination of results and implementation of interventions. Central is the commitment both to improvements in the community and to research [13, 14].

One such method is Group Level Assessment (GLA), which is a structured participatory research methodology for a large group to collaboratively bring forward and evaluate relevant needs and perspectives. In seven steps, community members generate, analyse and prioritize data together [15, 16].

With similarities to other large group stakeholder methods such as community summits [17], World Cafe’ [18], design charrettes [19], and search conferences [20], GLA allows the influence of researchers/field workers to be tuned down in favour of impact from members of the community with the process ideally resulting in participant-driven data and relevant action plans [16]. GLA has been used in a variety of community, hospital, social care, and educational settings to achieve multiple research objectives including needs/resource assessment, program evaluation, quality improvement, intervention development, feasibility/acceptability testing, knowledge generation and prioritization [21–26]. GLA stands in contrast to more traditional qualitative research methods which tend to be researcher-centric with researchers collecting and analyzing data provided by participants [15, 16]. Each step of the GLA actively and collaboratively involves a large group of non-formally trained researchers (in this case community members) in the generation, interpretation, and synthesis of data through a structured yet flexible process which culminates in the community prioritizing salient themes toward action [15, 16]. The outcomes of GLA include actionable ideas that are relevant to the target issue and encompass the collective view of the community members [22]. As a participatory research method, GLA aligns well with the guiding principles of CBPR and other community-engaged research approaches which emphasize shared decision making, co-learning, and shared leadership with community members, genuine and active collaboration between community members and researchers/professionals, and assets of the community [27]. Participatory research methods like GLA not only amplify the voice of community members but also position the “insider” lived expertise of local knowledge, norms, cultures, and contexts as equitable to the research expertise [10].

The aim of this study was to collaborate with residents living in a Swedish underserved residential area to generate health and wellness priorities and actions. We were particularly interested in issues related to dental health and nutrition. Specific research questions were: How can GLA facilitate reflections and actions grounded in the community? Which barriers and facilitators for a healthy life do inhabitants identify?

## Methods

### Setting and participants

Gårdsten is an underserved residential area located in the northern part of Gothenburg, Sweden and is home to a large number of recently arrived immigrants and refugees. Many have low income and limited formal education. In Gårdsten, caries and obesity are more prevalent than in the city as a whole, as are many other adverse conditions and illnesses [28, 29].

For the duration of this project, we formed a community-academic partnership composed of Gårdsten residents and researchers. The partnership worked collaboratively to make decisions and guide the work at each stage of the research process. The roles and responsibilities of this community-academic partnership are described in Table 1. The municipality approved the project and we used premises of the city of Gothenburg. Furthermore, we collaborated with the personnel at a public meeting place in Gårdsten.

**Table 1:** Roles and responsibilities of community-academic partnership in Gårdsten, Gothenburg, Sweden

**The community-academic partnership**	
The “participants” and “residents” refer to people living and/or working in the area who chose to participate after having been invited.	
“The community” refers to the neighborhood Gårdsten.	
“We” refers to the research group (i.e. people paid by the University of Gothenburg or the Angered hospital for this purpose and/or academic researchers providing research expertise). The research group consisted of five researchers (the authors) and two health workers with interest in health promotion, nutrition and odontology. We are women of different ages and two have worked in Gårdsten for many years but no one has lived in Gårdsten.	
**Shared responsibilities**	
***The research group:*** The project was initiated and organized by researchers and health care workers. We took initiative with the project, designed the research plan, organized and coordinated meetings, provided food, hired intercultural mediators, clarified the roles and responsibilities within the project, introduced and explained the participatory method, set the purpose for initial discussion, tried to facilitate trust and equity, moderated discussions, facilitated networking with stakeholders, and documented the process and the outcomes with transparency.	
***The participants/residents:*** The content of the project was driven by the participants/residents. They decided to take part in the project, invited other participants, commented on the design, reflected, discussed, gave their views, negotiated, interpreted and brought together various ideas and suggestions, and took responsibility for initiating, organizing, developing and driving actions emerging from the project.	
***Together:*** Striving for open, honest and fair-minded communication.	
**Perspectives on power relations**	
We reflected as suggested by Wolf [30] and Muhammad and colleagues [31] on the effect that our positionalities had on the power relations in the decision-making, processes, knowledge creation, publication and representation of voice. Cautious preparations were made of what to say and suggest in order to promote a trustful cooperation and avoid stigmatization. The responsibilities of individuals and groups were discussed with the participants throughout the project and we made clear that the research group acted as facilitators, and that we did not have any resources to finance solution of problems on behalf of the community. We strived to play down our own voices and give the floor to the participants, and at the same time take full responsibility in facilitating constructive participation, reflections, negotiations and actions. We made clear that our area of expertise and interest were within dental health and nutrition but that our intention was to facilitate any discussion related to well-being and health.	

The project started with two groups of Gårdsten residents, one during daytime and one in the evenings according to the participants’ preferences. At the first session, 24 and 15 adults respectively took part. After two meetings in each group, the residents decided to merge into one afternoon group. The groups were composed mostly of women. All those who came to the sessions were informed about the aim of the study, confidentiality and voluntariness and gave their written informed consent to participate. All methods were carried out in accordance with relevant guidelines and regulations.

### Data collection and procedure

Residents of Gårdsten met in a house open for the public located in the middle of the housing complex to participate in the research project. Residents were recruited through posters and postcards displayed at the housing complex and then by snowball sampling where residents invited neighbours and friends personally or by postcards distributed and posted on notice boards (e.g. staircases in the apartment buildings).

Since many languages and cultures were represented in the groups, and many residents did not speak fluent Swedish, we engaged intercultural mediators to attend each meeting to interpret and explain, and for one of the meetings also sign language interpreters. At each group meeting, 2 to 6 members of the research group were present to organize and moderate the discussions and take notes. The researchers had the intention to be facilitators of engagement as well as good listeners gathering information. Great effort was made to make the process transparent and to contribute to a trustful and equitable setting. At every meeting, food or snacks and beverages were provided and childcare if needed.

Following the steps of GLA methodology [16], the researchers facilitated group sessions over a period of 5 months for the participants to define their situation, identify priorities and plan actions for change. GLA proceeds through seven steps ─ climate setting, generating, appreciating, reflecting, understanding, selection and action and typically occurs in 1–2 sessions. In this case, the seven steps of GLA were extended to guide the research process over multiple sessions and groups attempting to sustain engagement of Gårdsten residents.

During these GLA sessions, members of the research team carried out a participatory observation and documented the discussions and the development and implementation of action plans in order to describe the process and the results.

At the first GLA session in each group, which lasted for 4 h, the participants mapped the situation in Gårdsten, reflected to understand each other’s perspective and discussed common views and desires (GLA steps 1–5).

**Step 1, Climate setting:** We started the first session by sharing a meal, doing introductions of all that were present, and describing the goals, objectives, and methodology of the project.

In **Step 2, Generating**, the participants were asked to answer pre-written, mostly open-ended, prompts related to health and wellbeing (Table 2) written on 20 flip charts placed on the walls in two rooms and a corridor. The participants moved around randomly to respond to prompts-- that is the prompts were not placed in a set order for them to follow. The research team members and intercultural mediators were available to support with translating, explaining and writing.

**Table 2:** Prompts for the first GLA session with residents in Gårdsten 2017

I wish everyone knew this about Gårdsten …			
What makes us feel good in Gårdsten is … .			
It is difficult to eat healthy because …			
In Gårdsten it is easy to …			
			
- → eat well			
- → move your body			
- → get rest and sleep			
- → meet other people			
- → shop good food			
In Gårdsten this is:			
good for health …	bad for health …		
A superhero for health in Gårdsten could …			
Children and young people in Gårdsten would need …			
What is required for children to be satisfied with their body?			
The school can support a good life Gårdsten by …			
Women/Men in Gårdsten would feel better if …			
Women …	Men …		
Elderly in Gårdsten would need ...			
Parents in Gårdsten would need …			
If we who live in Gårdsten got a billion SEK, we would use them for …			
Companies can support a good life in Gårdsten by …			
Associations can support a good life in Gårdsten by …			
It’s easy to get to …			
			
- the doctor			
- the dentist			
- the social service			
- the authorities			
- the Social Insurance Agency			
- the police			
Teeth are important for your well-being			
			
If Gothenburg was a city in a fairytale, how would you describe that city?			
Once upon a time there was a city which …			
If Gårdsten was a mountain in a fairytale, how would you describe the mountain?			
If I was allowed to change something in Gårdsten, it would be ...			

In **Steps 3–4** the participants read the responses, had the opportunity to add new comments or mark their agreement to other’s comments (**Appreciating),** and spend time to reflect on what the data as a whole meant to them **(Reflecting).**

In **Step 5, Understanding,** the participants divided themselves into small groups of 4 to 8 people according to language spoken. The research group helped to divide the flip charts among the groups according to which prompts they were interested in discussing and so that each small group had 4 to 7 charts. These small groups were instructed to, from their perspectives, interpret, discuss and identify 3–5 common and important themes across the charts. In each group, representatives from the research group were available to, if needed, facilitate discussion and record the final themes on a new flip chart. All groups were then gathered to discuss their results. A participant or facilitator from each small group then reported their themes and any insights regarding the themes. The results from each small group and the overall results were discussed by the larger group and were complemented with a few new themes raised from the discussion or because the large group found that important aspects from the individual comments had been ignored.

**Step 6, Selection:** In the second GLA session in each group, the discussion continued and the participants reflected on different perspectives, evaluated the themes, identified priorities, and began to develop goals and possible actions. The themes and goals in the evening and daytime groups were very similar. When the two groups merged into one group, data were further discussed, ideas were prioritized, and three common goals were formed.

**Step 7, Action:** In the third GLA session, the discussion was focused on how to reach the goals. Furthermore, a new common goal was decided among the group ─ to present the results of the discussions for key stakeholders (i.e. describe the situation in Gårdsten and the group’s suggestions for improvement). The participants also discussed which stakeholders to address and how to reach them. Two actions to reach this goal were discussed, to make an exhibition and a report. The participants were offered support with this, and it was decided that the research group should do writing and layout based on the participants’ results and suggestions. The participants invited other residents and other stakeholders to the exhibition and disseminated the report. For other future efforts, an action group emerged, consisting of four committed Gårdsten residents who wanted to continue work after the exhibition.

**Fig. 1:**
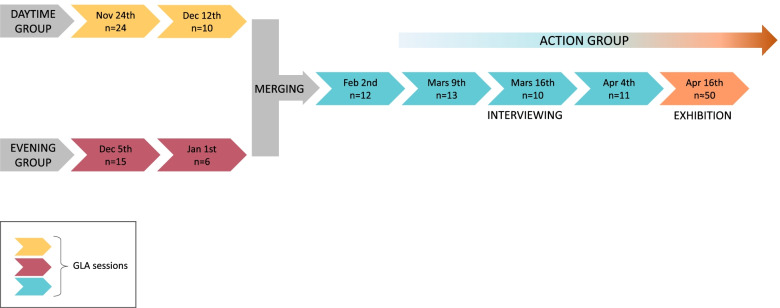
Flowchart of research project. An action group emerged during the sessions and continued after the research project with residents in Gårdsten. n = number of participants (children, intercultural mediators and researchers not included)

In a later GLA session (“Interviewing”, see Fig. 1), the researchers collected additional data by interviews to further clarify themes. This was initiated by some of the participants who wanted to describe their situation in Gårdsten to people outside. In six short interviews eight individuals, either alone or in pairs, i shared ideas related to the common themes and goals identified in the GLA sessions with one of the researchers. The researchers took detailed notes during these narratives. Passages and ideas that interviewees thought especially important were written down verbatim by the researchers. In the final GLA session, the research group presented the results that they had compiled for the exhibition and read all the quotes aloud. Participants approved the presentation with small revisions. We documented feedback from this session, combined it with the interview notes and included them in the report and the exhibition. At the final session, the details of the exhibition were discussed including who to invite and how best to reach people. We also discussed future work and roles moving forward. We made clear that the participants had the responsibility for coordinating and driving the future work, but that the research team was there to support them and facilitate their networking with stakeholders as needed.

### Data interpretation and analyses

Over several GLA sessions, the participants themselves generated and analysed the data from the flip charts and from summaries from previous discussions [15, 16]. In an iterative process where participants identified patterns in the responses across 4–7 wall charts (Step 5, Understanding), they reflected on the situation and the needs in the neighbourhood, and negotiated important themes on which to focus. Thereafter they discussed goals and options to reach the prioritized themes. In addition to being a support in this process, the researchers compiled the results from the different groups and summarised the information. We also took field notes from all GLA sessions. These were used to describe the process and the discussion leading to the themes, goals, and actions. The narratives from some of the participants illustrate the discussion about the situation in Gårdsten and were not further analysed.

This interpretation was grounded in the belief of participants’ competence, lived experience, and willingness to describe their situation as they experienced it. The standpoint was that our knowledge about life in Gårdsten was superficial and fragmentary, and therefore we consciously strived to understand issues from insider perspectives. We introduced the participants to the GLA steps and our role as facilitators of discussions and actions but gave no training in research methods. Thus, we gave no instructions about how to perform a thematic analysis, but rather emphasised the importance of prioritizing themes and goals that were important to them within the structured GLA process.

## Results

We conducted eight GLA sessions over a period of 5 months. In total, 47 Gårdsten residents participated in the sessions.

### Reflections about the situation and desired changes

Three overarching themes were identified by Gårdsten residents: 1) pride in Gårdsten yet need for improvement; 2) adolescent and parent needs for support; and 3) the importance of communication and meeting places. Although we had specific prompts related to dental health and nutrition, these topics were not central in the discussions.

#### Pride in Gårdsten yet need for improvement

Gårdsten was depicted by the participants as a nice place to live in, with beautiful surroundings and nice people who care for each other. Two friends described Gårdsten like this:

*"Everything is great! The best thing about Gårdsten is those who live here. We have lived here for a long time and like it."* (Interview 1, woman and man)

However, residents disliked how the area was portrayed in mass media. This is illustrated by a quote from one woman:

*“Media describes Gårdsten as a problem area. I'm so tired of that. That's not how it is. There are, of course, a few criminals. But there are only a few.”* (Interview 2, woman and man)

Even if Gårdsten mainly was described with pride by the participants they also emphasised two negative aspects. One was problems with littering and garbage disposal. The discussion was about “the other” not having knowledge and taking responsibility. Blaming other groups was an element in this discussion about littering, while most of the other reflections were built on the community as a whole. The other problem mentioned was that public services and facilities are closed down or relocated to other areas. They expressed a feeling of being abandoned by society. This was summarized as

*“Everyone moves away - only we, the poor ones, are left.”* (Quote from a discussion)

#### Adolescent and parent needs for support

Responses to the prompts and the subsequent discussions stressed adolescent and parent needs for support. Support to revitalize parents’ night patrols (i.e. people walking the neighbourhood to increase safety by being available and by marking the presence of adult society) and extended possibilities for children’s homework assistance were mentioned by the participants. In addition, residents emphasised the need for improved collaboration between schools and parents. Language and cultural differences were described as barriers to feeling supported in Gårdsten. Children acting as interpreters for their parents was viewed as a failure, and parents described the need for supportive interpreters who can act as liaisons with the school specifically. Two quotes illustrate this:

*“A young girl threw away all information received from the school. She was not doing well in school. It went bad. Those at school booked time with her mother. They told the mother that she did not manage school. The daughter interpreted and said: “Your daughter is good at school!” The mother smiled. They saw that she did not understand and explained that the daughter did not receive any grades. The daughter said to her mother: “She has top grades!” The mother smiled and appeared even happier. The school staff understood that something was wrong. They called for an interpreter. The mother cried and cried. Was very sad. Her daughter had been lying. You should not allow children to interpret for their parents*!” (Interview 3, woman)

"*The school writes [messages] to parents and their child throws them … One child did not go to school for a year - sold drugs. The school wrote letters to the parents, which their children interpreted. They signed even though they did not know what. The mother knows nothing. Very common."* (Interview 4, woman).

#### The importance of communication and meeting places

Emphasising communication as essential to health and wellbeing of their families, residents who did not have Swedish as their native language were eager to improve their Swedish skills. They wanted time and places to practice. For women with small children, it was hard to find these opportunities. Residents expressed desire for public meeting places for practicing Swedish where they could bring their children. One woman described that both parents and children need places to meet:

*“We want to meet others. You can do that at a meeting place. Communicate with others. Swap ideas. Get to know different cultures. Will then be easier to integrate into the Swedish society. Easier to exchange experiences. Those who have lived longer here can share experience with others. The children also get to know the society in which they will grow up.”* (Interview 5, woman)

In addition to emphasising the importance of learning Swedish, the difficulties of understanding information and the Swedish society in general were highlighted by participants. Residents said to achieve wellbeing they needed easily accessible information about many aspects of life in different languages. Residents also expressed the need for multiple information formats including written text, pictures, word of mouth, personal encouragement, and invitations. The need for receiving adequate information was described as a matter of safety. One of the participants expressed it like this.

*“You need information in your own language so that you understand 100%. Otherwise, you only understand some. You have to understand everything that says. It will then be easier to understand systems, society, care... It is not the same here as where we come from. The communities are very different. Everything is new; medical care, healthcare centers, the Social Insurance Office. All! Communities are different. If you get the right information, you will be safer.”* (Interview 6, woman)

### Suggestions, goals and actions

Based on the priority themes, the participants set four targets of action in Gårdsten:

- to advocate for better communication,
- to present their experiences, feelings and suggestions in a public report and an exhibition,
- to start a language café,
- to arrange a clean-up day.

An overarching goal was to advocate for better and various ways of communication. In addition to the specific suggested actions below, this was a future mission for themselves and others. They wanted better communication in the neighbourhood, for example, with the housing company, among the residents in Gårdsten and in the society in general. Improved information from and access to authorities was considered important. A qoute from a woman illustrate this:

*“Information in different languages is needed to be able to look for a job, social insurance, employment service, school.”* (Interview 4, woman)

According to the wishes of the participants, the research group took responsibility for the writing of a report and the arrangement of an exhibition where communication was one of the main themes. The content of the report and the exhibition was developed together with the participants. All texts were approved by the participants and the report was summarized in Arabic and Somali. The participants identified stakeholders to receive the report and to invite to the exhibition. Stakeholders included residents, housing company, representatives of the city, the local health centre and school, politicians and local associations. The report was disseminated within Gårdsten in both printed form and electronically by residents participating in the project and by stakeholders in the municipality. The researchers disseminated the report in their networks, which included local public health officials and politicians of the city. In addition, it was available at several public places in the neighbourhood.

The purpose of the exhibition was to gather strength to be able to continue work for a language café, more communication and future changes. Places where people can meet and practice Swedish were ranked as a high priority. It was considered as an important measure in the struggle against isolation, segregation, and communication barriers. One woman expressed it like this.

*“A language cafe is good. There I can practice the language and get in touch with other people. Good to communicate. Good to practice Swedish. At the same time, I can use my own language. You become less isolated. Several women are isolated.”* (Interview 5, woman)

Participants formed an action group with the goals to inquire about the requirements and to find potential collaborators for a language café in or near Gårdsten. The work of this group resulted in weekly open meetings run by some of the participants in cooperation with the Red Cross, hosted in the premises of the municipality. When planning this action one man argued for two necessary steps.

*"One: The first needed is a place where it is possible to do long-term work.*

*Two: Engaged people - Fiery souls. Commitment must come from the heart."* (Interview 2, man and woman)

The suggestion to arrange a clean-up day was not put into action. This issue was an expression of different needs from the residents (e.g., some wanted to meet and discuss with children and young people how to take care of surroundings, others hinted that certain “others” (lingual and cultural groups) were not keeping the area clean). Thus, the reason for suggesting a clean-up day was not only a matter of taking care of the environment, but also a way to meet and communicate with other people in Gårdsten. Participating residents decided to abandon the clean-up day since they learned that such clean-up days are arranged several times a year by the housing company although this was not known to all participants. One woman described that she had been invited but not have had confidence to take part:

*"I have also received an invitation to a cleaning day. That we would clean the yard together. I did not dare to go. I'm not very brave. Would need someone you know to go there. A friend or neighbour.”* (Interview 5, woman )

### In the aftermath of the GLA sessions

After the report was disseminated in the community, additional education about health, nutrition, and dental care was requested by visitors, including not only the original participants, at the public meeting place. When this need was expressed, the research group enabled the education by connecting dieticians and dental hygienists working within public child health care services with the visitors of the public meeting place. This resulted in several meetings with information shared and discussion about healthy food and teeth. Additionally, the research group was invited to a youth recreation centre in the area to conduct similar GLA sessions. The initial response was very positive, and the participants gave some reflections individually and as a group, but we did not reach the action phase. One of the main themes for the youth was the importance of politicians knowing about the less-than-ideal situation in Gårdsten. Another was mixed feelings toward the presence and behaviour of the police in Gårdsten and other areas in the suburb. Some youth expressed that they felt safer when the police were around, while other youth described that police were rude, checking on people “just because we live here.”

## Discussion

### GLA facilitated fruitful discussions and actions

GLA helped Gårdsten residents identify what they thought valuable and relevant concerning health issues and supported them in taking actions to achieve change. According to them, support to adolescent and parents, better community information and activities facilitating interpersonal interactions should be prioritized in Gårdsten. The GLA method worked to create open-ended discussions where perspectives from residents were put at the centre. Allowing for individuals to get support from intercultural mediators, the method also facilitated for participants with other mother tongue than Swedish to formulate their views.

The GLA methodology has previously been applied in diverse settings with specific groups of stakeholders [21–24, 26]. In the present study, GLA was used in a community setting with a less homogeneous group and with the more general objective to explore if it can be used for health promotion with people living in a particular residential area. Our results show that the method supported empowerment in that it could facilitate identification of important health issues and actions among involved residents in the community. Each of the steps of the method worked as planned, including the last one, Action. For instance, the resulting language café functioned for more than a year after the initial meetings.

Despite language barriers, the GLA discussions were open, vigorous and sometimes passionate with participants sharing information confidentially. The GLA-process facilitated reflections and dialog among the participants. The prompts inspired individual reflections and seeing others’ written comments expended those reflections and stimulated discussions.

### The GLA methodology could be expanded to give a deeper picture

Some of the women participants described themselves as isolated with many barriers for communication. They expressed that they wanted to convey their views of the situation in the community to other stakeholders and people outside Gårdsten. As researchers, we felt an obligation to make this happen but considered the data from GLA to be insufficient for depicting a nuanced and in-depth description. Therefore, we conducted interviews with eight additional participants who each shared personal narratives. These individual portrayals of the situation in Gårdsten illustrated the GLA sessions. Thus, the combination of the GLA sessions, resulting themes and additional interviews were successful in representing lived experience in Gårdsten. Our findings were supported by reflections from the participants and other residents when the report and exhibition were developed and launched.

### Consideration of power and community’s perceived needs

At first glance, the intervention may seem to have failed to fulfil its health promoting intentions since the themes and goals emanating from the participants did not concern nutrition and dental health. However, it is rather pointless to inform people about health issues in which they are not interested. Rather, an important conclusion is that health workers should discuss perceived needs with those concerned, endeavouring to get a trustful climate, before moving to action.

The fact that neither nutrition nor dental health were prominent topics in the participants’ discussions or among their proposals for health promotion indicates that the efforts to play down our own perspectives were successful. Thus, it seems that it was possible for the participants to take the lead and set the agenda based on their own priorities. It is also interesting that when the process had continued for some time, i.e. trust had started to build, residents asked for our expertise concerning nutritional and oral health. Since the research project was anchored in the established local public health network, it was possible to follow up on these requests and share the educational information within regular health services.

In the research group, we have had continuous discussions regarding power relations with deliberate attempts to ensure equity between us as researchers and residents of Gårdsten. Questioning researcher power and privilege has been emphasised as a fundamental element of forming collaborative partnerships with communities in the pursuit of health equity [31]. Wacquant’s [5] description of some neighbourhoods as being “increasingly perceived by both outsiders and insiders as social purgatories … where only the refuse of society would accept to dwell” (p 67) is relevant, and to internalize such a view of the place where you live is harmful to central aspects of health (i.e., self-efficacy and the sense of belonging to society). We were aware of the risk of adding to this stigma by acting as if people in the area are weak and vulnerable. Naming the community in this paper is a confirmation of the pride that residents, in spite of presented problems, felt for their neighbourhood and the contrasting picture (as compared to majority society’s) of it that they sketched out in the discussions.

Many actors are working to make Gårdsten a better area in which to live, but it may be challenging for them to balance this with governing responsibilities. One example is the Municipal Housing Company Gårdstensbostäder, which owns and manages the majority of all dwellings in Gårdsten. This public company has initiated several health promoting actions in the neighbourhood, but has also been described to create a relationship of dependence adding to the dominance that the residents are subject to by other parts of the society [32].

### Efforts and strategies to promote health literacy and trust

Many good health promotion initiatives exist [33, 34], but social institutions and other societal actors need to become more health literate. For this, it is necessary to develop new strategies and measures. Intercultural mediators and other professionals and volunteers with the mission to bridge between people, groups and institutions could be key persons [35]. Working *with* people, not on them, should be paramount [10, 36].

Trust is an important factor for equitable and sustainable societies and a prerequisite for successful health promotion and education [37–39]. Trust requires time, will and long-term planning. All social planning should be done from the outset that you do not risk damaging it. Pieces of the puzzle that need to be connected in order to make people’s everyday life work, like neighborhoods, housing companies, schools, interpreting services, social services and health care need to develop trust among themselves. Authorities who trust their residents dare to invite genuine community participation that enables ordinary people to have influence on important issues. This, in turn, may increase people’s trust in society. Most of the participants in this project seem to have opposite experiences, describing a lack of trust both with the authorities and with other groups of people living in the area. The quote “Everyone moves away - only we, the poor ones, are left” illustrates the view of a forsaken community where access to social institutions, shops, services and other facilities are limited in contrast with more affluent parts of the city.

### Limitations and strength

In spite of efforts to create an environment with equitable inclusion of all Gårdsten voices, it is likely that power relations affected the outcomes of this project. The participants who “had the floor” in the large group discussions and who led the action plans were the ones who already were somewhat engaged socially and comfortable using the Swedish language. It is likely that other issues would have emerged if all participants had been comfortable using Swedish language and knowledgeable regarding how to engage in civil society in the Swedish context. It is a known problem that those with greater relative power are able to articulate their views and take control, while the most marginalised have difficulties in expressing their interests and needs and taking part in community empowerment initiatives [40]. However, some of the writing and language barriers were overcome because of the nature of the GLA process and the cultural mediator bridging skills. Even if some participants did not take an active part in starting the language cafe’ or the other action steps, they were still able to make their voice heard individually and in smaller groups during the GLA sessions. We decided not to record the GLA-sessions, to avoid disturbing the reflections and discussions. Therefore, it was not possible to conduct deeper analyses beyond those of the participants. Likewise, detailed demographic information could not be presented since collection of such data might have influenced the participatory process by changing the focus and relations in the partnership.

No financial compensation was offered the participants (except for those who were employed at the meeting place or as intercultural mediators). There was no obvious impact of this imbalance, but we concluded that it would have been better to have a plan and financial means to compensate people who were willing to engage. We did not think it would be ethically correct to make specific attempts to re-include people who stopped coming to the sessions, or to investigate their motives.

The research group had decided in advance to use GLA, thus adopting the role of “consultant” [41]. However, the process was directed by the participants and by the sequence of events.

## Conclusions

Given the isolation and lack of voice of many vulnerable communities, it is essential to develop strategies and methods to promote participation and empowerment within the research process, and to evaluate them. However, conducting research within a CBPR framework is resource intensive and requires careful attention and commitment to both community engagement and action as well as research. GLA offers a promising way forward. GLA helped Gårdsten residents identify what they thought valuable and relevant concerning health issues and supported them in taking actions to achieve change. Improved individual and societal communication and trust are considered necessary for health, wellbeing, and a good life. Arenas like the open meeting place and professionals like the intercultural mediators are important bridges between health care and people when striving for health equity.

## Data Availability

The collected qualitative Swedish data used and analysed during the current study are available from the corresponding author on reasonable request.

## Abbreviations

GLA: Group Level Assessment
CBPR: Community-based participatory research
